# From Arrow to evidence: health economics and occupational health in the Nordic context

**DOI:** 10.5271/sjweh.4326

**Published:** 2026-09-01

**Authors:** Jarno Turunen

Modern health economics traces its origins to the 1960s, when Kenneth Arrow, an American Nobel laureate in economics, argued that healthcare differs fundamentally from goods traded in competitive markets, thus requiring special institutions and policies (1). His core ideas ─ uncertainty in health, the need for insurance, information asymmetry between actors, and limits to rational decision-making when health is at stake ─ laid the foundations for what is now known as modern health economics. The idea that healthcare cannot function as a standard market due to uncertainty and asymmetric information extends beyond hospitals. In the Nordic countries, occupational health systems can be understood, considering Arrow’s work, as institutional responses to these market failures, adapted to the realities of work, production, and labor markets.

This early work emphasized that illness is unpredictable and individuals lack the knowledge required to make informed health decisions. In the workplace, this challenge is amplified. Workers are not only uncertain about whether and when they will fall ill, but they may also have limited ability to observe, assess, or interpret the risks they face on the job. Employers likewise operate under imperfect information and uncertainty regarding workplace hazards and their consequences, although they may sometimes face incentives that lead risks to be underestimated or insufficiently recognized. Exposure to physical hazards, chemicals, or psychosocial stress is often better understood by specialists, regulators, and researchers than by workers or employers themselves (2–4). As a result, workplace risks are difficult to incorporate into labor market decisions in the way assumed by standard economic models of compensating wage differentials, which posit that workers can be compensated for accepting job-related risks through higher wages. In practice, however, many occupational risks are not fully observable, not well understood, or may be considered unacceptable regardless of compensation, limiting the applicability of such models.

One perspective arising from Arrow’s work concerns the organization of healthcare. In the context of occupational health and safety, it is useful to distinguish between general healthcare services available to workers and occupational health services, which focus specifically on the relationship between work and health. Occupational health services provide preventive activities such as workplace assessments, exposure monitoring, health surveillance, and advisory support and, in some countries, also include curative healthcare. In Finland, occupational health services are organized through a statutory system in which employers are required to provide preventive services for employees (5). As a result, coverage is extensive and services are widely available across the workforce. Internationally, occupational health services are organized through a variety of models, including employer-based systems, social insurance arrangements, public health service provision, and mixed approaches (6). The Finnish system therefore provides a useful point of comparison. Beyond providing care and prevention, occupational health services also act as producers and brokers of information, helping to mitigate information asymmetries. Through workplace assessments, exposure monitoring, health surveillance, and systematic data collection, they partially address the informational limitations identified by Arrow.

In this sense, occupational health systems socialize work-related health risks through regulation while addressing information asymmetry through structured interventions. The strong emphasis on prevention over treatment is highly consistent with Arrow’s framework: reducing risk *ex ante* is often more efficient than treating illness *ex post*. At the same time, occupational health in the Finnish model is not only a matter of welfare policy; it is also a labor market policy and productivity strategy. Whether these goals are achieved is ultimately an empirical question.

From the 1990s onwards, health economics and related fields have increasingly shifted towards modern econometric approaches aimed at credible causal inference (7). Techniques such as difference-in-differences, regression discontinuity designs, and instrumental variables have become central tools for estimating the effects of policies and interventions (8). Put simply, econometrics asks, “what is the effect?” (7), while economic evaluation asks, “is it worth it?” (9). Both questions ultimately depend on causal information (10). This brings us to the central counterfactual question: what would have happened in the absence of the intervention? In the Nordic countries, this can sometimes be directly addressed using randomized controlled trials (11–13). Often, however, causal inference methods are used to reconstruct this missing counterfactual in observational settings (8, 14–16). Without a credible counterfactual, we lack a valid measure of impact and cannot reliably estimate the benefits of an intervention for economic evaluation.

This has direct implications for resource allocation, which ultimately depends on causal accuracy. Economics is fundamentally concerned with scarcity, particularly under constrained public budgets, and economic evaluation is used to rank policies and guide funding decisions. For example, an occupational health program that appears to reduce sickness absence may reflect selection effects – such as healthier or more motivated employees being more likely to participate – rather than a true treatment effect. Without credible causal evidence, economic evaluation cannot distinguish true effects from bias, risks overstating benefits, and may lead to inefficient allocation of resources.

The Finnish occupational health system illustrates how Arrow’s insight – that health-related risks cannot be efficiently managed by markets alone – extends into working life. While national arrangements differ considerably (6), occupational health systems around the world share a common objective: managing work-related health risks through collective institutions rather than market mechanisms alone. However, despite their widespread use and longstanding role in protecting and promoting workers’ health, robust causal evidence on the effectiveness of occupational health services remains limited. This institutional solution therefore comes with its own requirement: rigorous evaluation. As labor markets evolve (17) and pressures on healthcare resources continue to intensify (18), the challenge is no longer only how to organize occupational health, but how to demonstrate that it improves health and welfare. Credible causal inference and sound economic evaluation are therefore essential. Without them, occupational health risks becoming a matter of assumption rather than evidence.
